# Parental Knowledge and Attitudes Towards *Helicobacter Pylori* Screening in Adolescents: A School-Based Questionnaire Study Among Guardians of Junior High School Students in Yokosuka City, Japan

**DOI:** 10.1007/s12029-024-01082-y

**Published:** 2024-06-27

**Authors:** Hiroaki Saito, Taiga Uchiyama, Mikio Matsuoka, Toshihiko Kakiuchi, Yuichiro Eguchi, Masaharu Tsubokura, Yasuhiro Mizuno

**Affiliations:** 1 Department of Internal Medicine, Soma Central Hospital, Soma, Japan; 2 Department of Radiation Health Management, Fukushima Medical University School of Medicine, Hikarigaoka 1, Fukushima, Fukushima 960-1295 Japan; 3 Chuou Naika Clinic, Yokosuka, Japan; 4 Department of Pediatrics, Faculty of Medicine, Saga University, Saga, Japan; 5 Loco Medical General Institute, Saga, Japan; 6 Ma-ru Clinic Yokosuka, Yokosuka, Japan

**Keywords:** *Helicobacter pylori*, Screening, Cross-sectional studies, Health knowledge, Attitudes, Adolescent

## Abstract

**Purpose:**

*Helicobacter pylori* (HP) infection, a risk factor for gastric cancer, is prevalent in Japan. Consequently, some municipalities across Japan are implementing HP screening and treatment programs for adolescents. However, little is known about parents’ attitudes and awareness regarding HP screening for their children. This study aimed to elucidate parental perspectives on HP screening for their children and identify the factors influencing these attitudes.

**Methods:**

This study focused on the parents of first-year junior high school students in Yokosuka City, Kanagawa Prefecture, where an HP screening and treatment program had been implemented for adolescents. The survey questionnaire was distributed among parents in all 23 public junior high schools in Yokosuka City.

**Results:**

Among the 618 respondents, 86.4% supported HP screening for their children. Regression analysis identified sufficient knowledge about HP (adjusted odds ratio (aOR) = 5.80; 95% confidence interval (CI), 2.10–16.03) and being in their 40s (aOR = 2.25; 95% CI, 1.35–3.77) as significant factors influencing supportive attitudes. For parents favoring the screening, common reasons included perceiving it as a promising opportunity (53.2%) and considering the test necessary (44.0%). In contrast, those who opposed screening frequently cited it as unnecessary (66.7%) or believed that their children did not have HP.

**Conclusions:**

A significant proportion of parents in Yokosuka City, Japan, demonstrated a good understanding of HP and expressed a high level of interest in HP screening for their children. Further investigation of parents’ attitudes is essential for the effective implementation of adolescent HP screening programs.

**Supplementary Information:**

The online version contains supplementary material available at 10.1007/s12029-024-01082-y.

## Introduction

Screening plays a crucial role in preventive interventions by facilitating early disease diagnosis and treatment in apparently healthy individuals. This strategy is particularly effective in mitigating the long-term consequences of chronic infections, such as hepatitis B and C viruses, tuberculosis, and *Helicobacter pylori* (HP) infection, all of which can lead to severe disease conditions and even cancers, if left untreated [1–3]. HP eradication has been shown to reduce the recurrence of gastric cancer, especially in regions with high incidence rates, such as Japan and South Korea [4–6]. Recent studies have suggested that HP eradication may also suppress the development of gastric cancer in asymptomatic adults [7, 8], leading to the implementation of screening and treatment strategies in high-risk areas, particularly in Asia [9, 10]. Thus, addressing HP infection and reducing the incidence of gastric cancer are crucial public health objectives.

In several countries, including those in Asia where the prevalence of gastric cancer is high, there has been a growing interest in screening adolescents for HP [11–15]. HP is believed to cause infection during infancy and to increase the risk of cancer over time by inducing metaplasia in the gastric epithelium. In Japan, the estimated cumulative incidence risk for gastric cancer from birth to 85 years in the infected population is reported to be 17.0% for men and 7.7% for women [16]. Eradicating HP before the progression to gastritis is thought to offer a potentially more effective preventive approach for gastric cancer [17]. However, there is a dearth of research on the optimal age for HP screening and eradication. Japan, with its high incidence and mortality rates of gastric cancer, has started conducting screening and treatment interventions for adolescents at the municipal level [12, 18–20].

Yokosuka City, an urban area in Japan, was an early adopter of the HP screening program for adolescents. The city initiated a pilot program for HP testing in junior high school students in 2017 and later introduced HP testing as a screening tool for all second-year junior high school students in 2019. This involved measuring antibodies in urine samples collected at school and performing a urea breath test at a medical institution if the outcome was positive. Subsequently, eradication treatment was provided to those who tested positive for HP. To date, approximately 10,000 students have participated in this program. Similar to adult screening initiatives, sustaining and improving primary testing participation rates continues to be a substantial challenge in these screening programs.

Adolescent health screening relies heavily on parents’ understanding and support. This element is evident in contexts like the human papillomavirus (HPV) vaccine, where an individual’s decision to participate in screening is influenced by their understanding of HPV and cervical cancer, as well as their parents’ perception [21, 22]. Likewise, comprehending a disease like HP entails not only recognizing it as an infectious disease but also grasping complex medical knowledge, including implications for potential future gastric cancer risk. Despite the importance of these factors, surveys exploring how parents perceive their children’s HP screening outcomes and their level of comprehension regarding the infection are lacking.

Our study aimed to clarify the key determinants affecting parental decisions regarding HP screening for their children and to evaluate parents’ current level of knowledge about the infection. To achieve this, we conducted a questionnaire-based survey of middle school students’ parents in Yokosuka City, Kanagawa Prefecture, focusing on their perceptions and understanding of HP and its related screening procedures. Through this study, we sought to identify effective methods to enhance HP screening programs for adolescents, address the identified issues to increase participation rates, and improve the overall effectiveness of these programs.

## Materials and Methods

### Participants and Setting

This study conducted a questionnaire-based survey of respondents for 5 weeks, from April 7, 2022, to May 13, 2022. The eligible population comprised 2793 first-year students enrolled in all 23 municipal middle schools in Yokosuka City, Kanagawa Prefecture. With the cooperation of the Health Management Support Division of Yokosuka City, and Yokosuka City Board of Education, the survey consisted of 15 questions and was administered via an online response platform. QR codes and URLs providing access to the survey were distributed at each middle school and were subsequently conveyed to parents by their children. Although the content was primarily in Japanese, non-Japanese-speaking parents were offered English language guidance, and response forms were also provided in English language. Participation was voluntary and informed consent was obtained upon completion of the survey. No specific monetary compensations or incentives were offered for completing the survey. Before implementation, this study was approved by the Ethics Committee of the Yokosuka City Medical Association (approval number 1) and Fukushima Medical University (approval number 2022-030).

### Questionnaire Content

The items in the questionnaire, which tested the knowledge of HP among parents, were developed with reference to previous studies [23, 24]. The survey items encompassed fundamental knowledge of HP, sources of medical information, awareness of HP screening among middle school students in Yokosuka, willingness to allow their children to participate in screening, and the reasons for their decisions. The questions related to knowledge regarding HP covered aspects such as harmful effects of HP, site of infection, mode of transmission, and details about testing and treatment. Respondents were also asked about their basic characteristics, including sex, family history of HP and cancer, history of HP testing/treatment, participation in cancer screening, occupation (medical or non-medical), the respondent’s relationship with the student, student’s sex, age, presence of siblings, and cohabiting family members (Supplementary file). Internal validity of the questionnaire was assessed by two gastroenterologists (HS and YM) and one internist (MT). To ensure anonymity, the survey did not collect data identifying individuals, and responses with missing data on the willingness to allow children to participate in HP screening were excluded from the analyses.

### Statistical Analyses

Statistical descriptions are presented as medians (quartiles) for continuous variables and as proportions for categorical variables. For knowledge regarding HP, respondents were categorized based on the number of correct answers to the following seven questions: insufficient knowledge (0–2 correct answers), intermediate knowledge (3-4 correct answers), and sufficient knowledge (5–7 correct answers). We conducted univariable and multivariable logistic regression analysis. The following factors, which seemed to influence the willingness of children to undergo HP testing [23], including the respondents’ age, sex, categorized HP knowledge, presence of HP in the family, family history of cancer, HP testing, and adherence to cancer screening, were included for the analysis. *p*-values of 0.05 or less were deemed statistically significant. Data analysis was performed using Stata/IC ver 15.1 for Mac (College Station, TX: StataCorp, USA).

## Results

### Demographics of Respondents

Of the 2793 eligible students, 618 parents responded, representing a response rate of 22.1%. Respondents’ demographics are presented in Table 1. Most of the respondents were mothers (90.4%) in their 30s and 40s (87.4%). Regarding medical history, 230 (37.2%) had undergone HP testing, 49 (7.9%) had received HP treatment, 124 (20.1%) reported a family history of HP infection, 153 (24.8%) reported a family history of cancer, and 43 (7.0%) had stomach cancer. In terms of medical information sources, 490 respondents (79.3%) referred to the Internet, 436 (70.6%) to television, and 278 (45%) consulted their primary care physicians (Supplementary Table 1).

**Table 1:** Characteristics of the participants and children

**Variables, *n* %**	***n* = 618**
**Respondents**	
** Age**	
30–39 y	148 (24.5)
40–49 y	380 (62.9)
50–59 y	73 (12.1)
60–69 y	2 (0.3)
70–79 y	1 (0.2)
No answer	14 (2.3)
** Relationship**	
Mother	549 (90.4)
Father	47 (7.8)
Grandmother	5 (0.8)
Grandfather	5 (0.8)
No answer	12 (0.2)
**Child**	
** Sex**	
Male	322 (53.1)
Female	285 (47.0)
No answer	11 (1.8)
** Siblings**	
Present	513 (83.0)
Younger brother	160 (31.2)
Older brother	174 (33.9)
Younger sister	159 (31.0)
Older sister	163 (31.8)
Absent	85 (13.8)
No answer	20 (3.2)
**Respondent’s HP testing history**	
Have experience of a prior HP test	230 (37.2)
Never underwent HP testing	382 (61.8)
No answer	6 (1.0)
Respondent’s HP treatment history	
Had received HP eradication therapy	49 (7.9)
Never received HP eradication therapy	559 (90.4)
No answer	10 (1.6)
Respondent’s cancer screening adherence	
Annual participation	154 (24.9)
Occasional participation	243 (39.3)
Never	215 (34.8)
No answer	8 (1.3)
**Family history of HP infection**	
Present	124 (20.1)
Grandfather	26 (21.0)
Grandmother	42 (33.9)
Father	29 (23.4)
Mother	32 (25.8)
Siblings	4 (3.2)
Others	7 (5.6)
Absent	479 (77.5)
No answer	15 (2.4)
**Family history of cancer**	
Present	153 (24.8)
Gastric cancer	43 (7.0)
Absent	465 (75.2)
No answer	20 (3.2)
**Respondent’s field of work**	
Medical	102 (16.5)
Non-medical	505 (81.7)
No answer	11 (1.8)
**Attitude towards HP screening for their children**	
Supportive	400 (64.7)
Somewhat supportive	134 (21.7)
Somewhat negative	7 (1.1)
Negative	5 (0.8)
Neutral	72 (11.7)

*y *years old,  *HP* *Helicobacter pylori*

### Respondents’ Awareness and Knowledge of HP

Table 2 presents the percentage of correct answers regarding knowledge of HP among the respondents. The correct response rates for questions related to the harmfulness of HP, sites of infection, modes of transmission, and family infection were 88.7, 84.1, 61.5, and 47.2%, respectively. Notably, 95.7% correctly identified the diseases caused by HP, and 85.6% knew that they could be detected during screening. Additionally, 90.3% correctly answered that HP could be treated with oral medications. Of the seven questions, 19 (3.1%) answered 0–2 correctly; 100 (16.2%), 3–4; and 499 (80.8%), 5–7. The choices for each item are presented in Supplementary Table 2. Regarding the preferred HP testing methods, 61.7% of respondents preferred blood testing, 38.3% preferred urine testing, and 31.1% opted for breath testing (Supplementary Table 3).

**Table 2:** Correct answer response rates for HP knowledge

**Topic**	**Correct answer choice**	**Number of respondents with correct answers**
Harmfulness of HP	Harmful	548 (88.7)
Site of HP infection	Stomach	520 (84.1)
Transmission route for HP	Hand-mouth transmission	379 (61.5)
Transmission among family members	Yes	291 (47.2)
What disease is caused by HP?	Gastric diseases (ulcer, cancer)	583 (95.7)
Can screening detect HP?	Yes	529 (85.6)
Treatment of HP	Oral medicine	557 (90.3)

Numbers represent the count of answers and proportions

*HP* *Helicobacter pylori*

### Parental Desire for HP Screening in Children

Among the 618 respondents, 534 (86.4%) expressed their support for HP screening of their children. Univariate logistic regression analysis revealed significant associations with supportive attitudes towards HP screening for children, which included sufficient knowledge of HP (odds ratio (OR) 6.25, 95% CI 2.41–16.2, *p* < 0.05), being in their 40s (OR 2.25, 95% CI 1.35–3.77, *p* < 0.05), family history of HP infection (OR 2.01, 95% CI 1.01–4.01, *p* < 0.05), high adherence to cancer screening (OR 2.32, 95% CI 1.19–4.54, *p* < 0.05), and being a healthcare professional (OR 2.33, 95% CI 1.04–5.22, *p* < 0.05) (Table 3). Multivariable regression analysis further confirmed the significant associations of sufficient knowledge of HP (OR 5.80, 95% CI 2.10–16.03, *p* < 0.05) and being in their 40s (OR 2.25, 95% CI 1.35–3.77, *p* < 0.05) with parental preference for screening. Since the number of respondents in the “insufficient knowledge (0–2 correct answers)” category, which was used as the reference category for knowledge level, was small, a similar multivariable regression analysis was also conducted using insufficient and middle knowledge (0–4 correct answers) as the reference category for knowledge level, and similar results were obtained (Supplementary Table 4).

**Table 3:** Variables related to the desire for children’s HP screening

Variables	Univariable regression analysis		Multivariable	*p*-values
	OR (95% CI)	*p*-values	Adjusted OR (95% CI)	
**Knowledge of HP** **(reference = insufficient (0–2 points))**				
Sufficient (5–7 points)	6.25 (2.41–16.2)	< 0.05	5.8 (2.10–16.03)	< 0.05
Middle (3–4 points)	2.30 (0.83–6.39)	0.11	2.25 (0.78–6.46)	0.13
**Relationship (reference = father)**				
Mother	1.62 (0.75–3.50)	0.22	1.56 (0.66–3.64)	0.31
Grandmother	0.95 (0.09–9.53)	0.96	0.62 (0.05–7.04)	0.70
Grandfather	0.95 (0.09–9.53)	0.96	0.88 (0.07–10.76)	0.92
**Age (reference = 30–39 y)**				
40–49 y	2.25 (1.35–3.77)	< 0.05	2.21 (1.25–3.89)	< 0.05
50 y	1.75 (0.81–3.79)	0.16	1.92 (0.81–4.54)	0.14
**Occupation (reference = other jobs)**				
Medical professional	2.33 (1.04–5.22)	0.04	1.64 (0.70–3.87)	0.25
**Family HP history (reference = absent)**				
Present	2.01 (1.01–4.01)	< 0.05	1.76 (0.82–3.79)	0.15
**Family cancer history (reference = absent)**				
Present	1.06 (0.62–1.82)	0.83	0.82 (0.45–1.49)	0.52
**HP screening history (reference = absent)**				
Present	1.21 (0.75–1.98)	0.44	0.64 (0.35–1.14)	0.13
**Cancer screening adherence (reference = never)**				
Occasionally	1.47 (0.88–2.46)	0.14	1.26 (0.71–2.26)	0.43
Annually	2.32 (1.19–4.54)	< 0.05	1.61 (0.76–3.41)	0.21

*OR* odds ratio, *CI* confidence interval, *HP* *Helicobacter pylori*

### Reasons for Parental Preferences Regarding HP Screening for Children

Regarding the reasons for wanting their child to undergo HP screening, the most common response was, “It is a favorable opportunity” (53.2%), followed by “HP testing is necessary” (44.0%), and “The cost is covered”, along with “Family members have undergone HP testing” (Supplementary Table 5a). Conversely, among the 12 respondents who did not prefer that their child should undergo HP testing, the most common reasons were “HP testing is unnecessary” (66.7%), followed by “Our child does not have HP”, “We are not familiar with *Helicobacter pylori*”, “Our child’s friends are not interested in the screening”, and “No one in the family has undergone HP testing” (Supplementary Table 5b).

## Discussion

In this study, we conducted a comprehensive survey to assess knowledge and attitudes towards HP screening among the parents of first-year middle school students in Yokosuka City, Kanagawa Prefecture, Japan. The findings revealed a high degree of understanding of HP among parents, with a significant majority indicating a desire to have their children screened. Notably, a positive correlation was observed between higher levels of knowledge and supportive attitudes towards screening for children. While it is important to note that the association between the level of knowledge of HP and desire for screening their children is not necessarily causal, this study may imply the importance of providing accurate information about HP to parents when offering HP screening for children. Further exploration of the reasons behind different attitudes towards HP screening is warranted. Future research should be focused on personalized strategies based on participation preferences, such as targeted educational campaigns, personalized communication, and involving healthcare professionals to address specific concerns and provide detailed information.

The knowledge levels of the participating parents regarding HP were notably high, with > 80% rated as having a good understanding of HP, which contrasts with previous surveys conducted in different countries. For instance, a study in a Singapore hospital involving an Asian population revealed that only 30.2% of respondents had a correct understanding of HP [24]. Similarly, a study in China on the general population reported correct answers for HP infectivity and prevention at 35% and 43.6%, respectively [23]. Another study involving multi-ethnic Asian students showed that only 30.2% of the participants were aware of HP [24]. The high level of knowledge observed among parents in our study can be attributed to the widespread availability of HP screening for adults in Japan, particularly through the ABC method, which measures HP antibodies and pepsinogen levels in the blood and is implemented in many municipalities in Japan including Yokosuka City [25]. The successful implementation of HP screening in both children and adults in the targeted region of this study might explain the higher level of parental knowledge of HP. Therefore, it is essential to not only to raise awareness but also to promote a correct understanding of HP as a preventive measure for gastric cancer and deliver information that meets the expectations of the general population in such regions.

To the best of our knowledge, this study is the first to investigate parental intention regarding HP screening in children. Interestingly, over 80% of the respondents expressed a desire for their children to undergo screening, mirroring the high level of interest observed in adult HP screening. In a study conducted on the general population in China, with a gastric cancer mortality rate of 15.9 per 100,000 persons [26], over 80% of participants expressed willingness to undergo HP screening for gastric cancer prevention [23]. Similarly, in Korea, 73.7% of the general population supported the “HP screen and treat” strategy [27]. It is widely acknowledged that lack of knowledge about HP contributes to negative attitudes towards screening [23, 28], which aligns with parents’ screening intentions for their children. Although not statistically significant after adjustment, a family history of HP infection, working in the medical profession, and a positive attitude towards cancer screening showed a positive trend for providing HP screening for children. These findings highlight the role of parents’ own experiences and engagement with HP, which may generate interest in HP-related issues and influence their willingness to screen their children. These insights are vital for planning HP screening for children, as regions with parents demonstrating sufficient knowledge, experience, and interest in preventive medical behaviors related to HP are more likely to be receptive to screening initiatives, whereas regions with low awareness may have less inclination for children’s screening. Therefore, a preliminary assessment of parental understanding and interest regarding HP in different regions is necessary when considering HP screening for children.

The most common reason cited among those who did not prefer that their child should undergo HP testing was the belief that the test was unnecessary, coupled with the perception that the children did not have HP. Such perceptions of HP infections have been frequently reported, even in regions with a high prevalence of HP, where a considerable proportion of individuals report not being infected or perceive a very low risk of gastric cancer [27]. Although the HP infection rate among Japanese teenagers is reported to be < 5% [15], it may contribute to such indifference or normalcy bias. To address these attitudes, future awareness campaigns and information provision should emphasize the importance and effectiveness of HP screening.

Conversely, among parents desiring HP screening for their children, the most common reasons were that it presented a favorable opportunity, screening was necessary, and that it was cost-free. These reasons indicate a positive understanding of HP and willingness to participate in screening, without any barriers or reasons to decline. It is evident that some individuals are comfortable participating in the screening, express a desire for it, and lack reasons to refuse based on their understanding of HP. In particular, it is worth noting that cost is known to be a barrier for undergoing any screening and that many respondents in this study said they would like their children to undergo screening because it does not cost anything. Yokosuka City pays for the cost of testing and treatment of HP for junior high school students in the city. On the other hand, tests for HP and screening tests for stomach cancer, which are generally offered to adults, incur costs. To ensure that those who convey a desire for screening receive it, it is critical to eliminate institutional barriers, remove participation obstacles, and minimize economic burden. Furthermore, providing a comprehensive explanation of the necessity of HP screening is crucial for maintaining a positive attitude towards participation.

Understanding the direction and reasons behind screening preferences is essential to effectively approach different groups with varying inclinations towards HP screening among adolescents [29]. For instance, the most common reason parents wanted their children to undergo HP screening was “It’s a favorable opportunity”, which may reflect the psychological tendency to choose the default option [30]. Additionally, the presentation of screening participation is crucial and can be influenced by framing techniques. In this study, the reasons for desiring screening included the absence of cost, avoidance of hospital visits, and test necessity. For these elements, the framing technique was used for adding emphasis, for example, “If you miss this screening opportunity, you will have to pay for the screening” or “If you miss the screening, you will have to visit a hospital.” Explanations highlighting losses may increase the willingness to participate, but whether gain framing or loss framing are useful is still uncertain for promoting the HP screening participation for adolescents. Further accumulation of cases would help in determining the optimal approach for individuals who do not wish to undergo screening, or whose preferences are undecided.

In terms of future implications, this study revealed a high interest among parents in having their children undergo screening for HP. However, a small number of individuals either declined to participate or expressed tentative attitudes towards it. Understanding the factors, health perspectives, and reasons of these individuals is crucial for efficient screening. Furthermore, there are regional variations in the provision of HP screening for adolescents in Japan. Therefore, conducting large-scale surveys in diverse regions would yield more meaningful outcomes. Additionally, specific issues related to adolescent screening, such as the psychological burden on individuals undergoing screening and parental attitudes, were not explored extensively in this study. We did not collect any information regarding the desire of children for participation in the screening. As for cancer screening in adolescents, the willingness of the children to undergo screening has been reported to be important [31]. Future research should address these differences by examining the challenges encountered in other screening programs, such as pediatric thyroid cancer screening, in which the psychological impact on individuals and parental attitudes has been discussed.

This study has several limitations. First, this study included a survey sample restricted to a single municipality in Japan, where HP screening was available for adolescents, potentially limiting its representativeness for other municipalities. Conducting a study that encompasses multiple municipalities would yield more robust results. Furthermore, the response rate of the survey was 22%, and the act of responding itself may indicate an interest in HP, potentially introducing bias and limiting the generalizability of the results to the entire population. For example, 14.1% of respondents in their 20s to 40s in Yokosuka City were reported to be healthcare workers [32], while 16.5% of the overall respondents in this survey were healthcare workers, which was slightly higher. It is possible that those interested in HP were more likely to respond. Therefore, the general public may have less knowledge related to HP than was obtained in this study. On the other hand, in order to collect responses from more general parents, this study was conducted with the cooperation of the Health Management Support Division of Yokosuka City and the Yokosuka City Education Board, in which parents were notified. Such a study testing citizens’ knowledge of HP is unprecedented, and we believe it will be of interest in future research. Hence, unbiased survey methods are warranted. Lastly, as the survey was conducted in schools in Yokosuka City, sensitive background information such as educational background and income could not be collected.

## Conclusion

The parents of middle school students in the study were familiar with HP and expressed a strong desire for screening their children for HP. Having sufficient knowledge was significantly related to the desire to have their children undergo HP screening. Further investigation of parental attitudes towards children’s screening is crucial for the appropriate implementation of screening programs.

### Supplementary Information

Below is the link to the electronic supplementary material.

Supplementary file1 (DOCX 30.2 KB)

Supplementary file2 (DOCX 32 KB)

## Data Availability

Data supporting the findings of this study are not publicly available. However, data are available from the authors upon request.
