# Supplementary material for: Parental Knowledge and Attitudes Towards Helicobacter Pylori Screening in Adolescents: A School-Based Questionnaire Study Among Guardians of Junior High School Students in Yokosuka City, Japan
Source: J Gastrointest Cancer. 2024 Jun 27;55(3):1274–81. doi: 10.1007/s12029-024-01082-y (PMC11347493; doi:10.1007/s12029-024-01082-y)
Supplement: Supplementary file 1 — Supplementary file1 (DOCX 30.2 KB) [file 12029_2024_1082_MOESM1_ESM.docx]

**English translations of questionnaires**

Please answer the following questions.

If you have questions that are difficult to answer or do not wish to answer, please do not answer them, and proceed to the next question.

Question1; regarding “Helicobacter. pylori.” Bacteria.

For each question, please choose the one you think is appropriate

Question 1-1

Do you know “*Helicobacter pylori* (*H. pylori*).” bacteria?

・I know it well.

・I know it.

・I have heard the name.

・I don’t know.

Question 1-2

What do you think is the site of the *H. pylori* infection? (multiple choices allowed）

・Brain

・Heart

・Small intestine

・Kidney

・Liver

・Stomach

・I don’t know.

Question 1-3

What do you think of when you hear the word "*H. pylori*"?

・Positive impression

・Negative impression

I do not have any impressions.

・I don’t know.

Question 1-4

How do you think *H. pylori* is transmitted?

・Oral infection

・From mother to child during pregnancy.

・Sexual infections

・Needlestick accidents involving syringes and other needles

・All of above

・I don’t know

Question 1-5

Do you think there is a chance of infection among family members living together?

・I think so.

・I don’t think so.

・I don’t know.

Question 1-6

What diseases do you think are caused by *H. pylori* infection?

・Brain infection

・Kidney failure

・Cirrhosis of the liver

・Pneumonia

Gastrointestinal symptoms (ulcers and cancer)

・Not causing any particular problems.

Question 1-7

Do you think *H. pylori* can be found in a medical screening?

・I think so.

・I don’t think so.

・I don’t know.

Question 1-8

If you were to be tested for *H. pylori* infection, what would you prefer? （multiple choice allowed）

・Blood test

・Stool test

・Urinary test

・Breath test

・Upper Endoscopy

I do not want to perform any of these tests.

Question 1-9

How do you think *H. pylori* can be cured?

・Oral medicine

・Intravenous drip

・Surgery

・Can’t be cured.

・I don’t know.

Question 2

What do you usually refer to as sources of information about medical care? Please select all that apply.

・Television

・Newspaper

・Radio

・Internet

・Social network services, such as Twitter, Facebook

・Magazine

・Books

・Stories of acquaintances and friends

・Information from your family physician

We would like to ask about the *H. pylori* screening for junior high school students in Yokosuka City.

Yokosuka City provides an opportunity for "*H. pylori* checkups" for second-year junior high school students.

Question 3

Have you heard about this project? Please select one of the following.

Yes, I have heard of this project.

No, I have not heard about the project.

Question 3-a

Would you be willing to have your child tested for *H. pylori* infection if notified?

Please choose one that applies.

I want my child to take the test.

I would rather have my child take a test.

・Neither

・Somewhat reluctant to let my child take the test

I do not want my child to take the test.

Question 3-b

If you answered, " I want my child to take the test." or " I would rather have my child take the test.". For Question 3-a, please answer the following question:

What are the reasons Why do you want your child tested? (Multiple choices allowed)

・None in particular

・Cost issues (free, cheap, etc.)

・An opportunity for testing was offered.

・Others around me are also taking it

・Because it is done at school

・I think an H. pylori test is necessary.

・Some members of my family were tested for H. pylori infection.

・(His or Her) siblings participated in the test.

・I do not have to go to the hospital.

・Others

Question 3-c

If you answered, " I don't want my child to take the test." Somewhat reluctant to let my child take the test " In question 3-a, please answer the following:

What are the reasons (multiple choice)?

・Nothing in particular

・Busy

・I do not think that an *H. pylori* test is necessary.

・I do not think my child has *H. pylori*

・Not sure about *H. pylori*

・People around me do not accept this.

・Because it is done at school

・No one in my family tested positive for *H. pylori*.

・Other

Question 4

What would you refer to regarding whether you would have your child test positive for *H. pylori*? (Multiple choices allowed)

・His/her wishes

・Participation of classmates

・Method of the test

・Cost of the test

・Advantages of the test

・Disadvantages of the test

・Current Symptoms

・Necessity of a hospital visit

・Family Doctor's Recommendation

・Whether family members have been tested for H. pylori

・Busyness of the respondent (parent/guardian)

・Other

Question 5

What would you like to know about *H. pylori* screening among junior high-school students?

・None in particular.

・H. pylori itself.

・How to test for *H. pylori* and the cost

・The Necessity of *H. pylori* testing

・Regarding the anti-*H. pylori* program

Results of previous screening projects

・Other

Question 6

Please respond to your comments regarding the *H. pylori* screening program for junior high school students.

Question 7

Please answer the following to the extent.

Sex of your child (1st year junior high school student)

・Male

・Female

Siblings of your child (first-year junior high school student)

・None

・elder brother (Please indicate the number)

・younger brother(Please indicate the number)

・elder sister (Please indicate the number)

・younger sister (Please indicate the number)

Family members living with the student

・Father

・Mother

・Siblings

・Grand father

・Grand mother

*H. pylori* status in your family

・No (unknown)

Yes ( student).

・Yes (Father)

・Yes (mother)

・Yes (brother/sister)

・Yes (grandfather).

・Yes (grandmother)

・Yes (Other)

Family history of cancer

・None

・Yes（indicate the type of cancer）

About the respondent of the survey

・Father

・Mother

・Grand mother

・Grand father

・Other

Age of the respondent

・20s

・30s

・40s

・50s

・60s

・70s

・80s

Please select the respondent's history of *H. pylori* testing.

I have undergone *H. pylori* testing.

・Never.

Please select the respondent's history of *H. pylori* treatment.

I have undergone *H.pylori* treatment.

・Never.

Have you ever had a cancer screening at work or in Yokosuka City?

・I take it every year

I have experienced occasional

・Never

The type of your occupation

・medical professionals.

・Others.

**Thank you for your cooperation.**
