# Supplementary material for: Parental Knowledge and Attitudes Towards Helicobacter Pylori Screening in Adolescents: A School-Based Questionnaire Study Among Guardians of Junior High School Students in Yokosuka City, Japan
Source: J Gastrointest Cancer. 2024 Jun 27;55(3):1274–81. doi: 10.1007/s12029-024-01082-y (PMC11347493; doi:10.1007/s12029-024-01082-y)
Supplement: Supplementary file 2 — Supplementary file2 (DOCX 32 KB) [file 12029_2024_1082_MOESM2_ESM.docx]

**Supplementary Information**

**Parental Knowledge and Attitudes towards *Helicobacter Pylori* Screening in Adolescents: A School Based Questionnaire Study among Guardians of Junior High School Students in Yokosuka City, Japan**

***Journal of Gastrointestinal Cancer***

Hiroaki Saito^1,2*^, Taiga Uchiyama^1^, Mikio Matsuoka^3^, Toshihiko Kakiuchi^4^, Yuichiro Eguchi^5^, Masaharu Tsubokura^1^, Yasuhiro Mizuno^6^

**Affiliations**

^1^Department of Internal Medicine, Soma Central Hospital, Soma, Japan

^2^Department of Radiation Health Management, Fukushima Medical University School of Medicine, Fukushima, Japan

^3^Chuo Naika Clinic, Yokosuka, Japan

^4^Department of Pediatrics, Faculty of Medicine, Saga University, Saga, Japan.

^5^Loco Medical General Institute, Saga, Japan

^6^Ma-ru Clinic Yokosuka, Yokosuka, Japan

***Corresponding author:**

Hiroaki Saito

Email: hiros@fmu.ac.jp

**Contents:**

**Supplementary Table legends:**

**Supplementary Table 1: Source of knowledge on medical issues**

**Supplementary Table 2: Detailed response rates to questions on *Helicobacter pylori* knowledge**

**Supplementary Table 3: Acceptable tests for *Helicobacter pylori* screening**

**Supplementary Table 4: Variables related to the desire for children’s *Helicobacter pylori* screening**

**Supplementary Table 5a: Reasons for guardians supporting screening of children**

**Supplementary Table 5b: Reasons for guardians not supporting the screening of their children**

**Supplementary Table 1. Source of knowledge on medical issues**

| **Source of knowledge** | **Number of answers (n=618)**  **n (%)** |
| --- | --- |
| Television | 436 (70.6) |
| Newspaper | 85 (13.8) |
| Radio | 28 (4.5) |
| Internet | 490 (79.3) |
| Social network service | 99 (16.0) |
| Magazine | 54 (8.7) |
| Books | 108 (17.5) |
| Peers | 233 (37.7) |
| Family physicians | 278 (45.0) |
| No answer | 5 (1.0) |

The numbers represent the count of answers and the proportion.

**Supplementary Table 2. Detailed response rates to questions on HP knowledge**

| **Topic** | **Number of respondents (n=618)**  **n (%)** |
| --- | --- |
| **Knowledge of HP** |  |
| Know well | 93 (15.1) |
| Know | 333 (53.9) |
| Don’t know | 6 (1.0) |
| I have heard of it | 186 (30.1) |
| **Harmfulness of HP** |  |
| Bad impression | 548 (88.7) |
| Neutral | 42 (6.8) |
| Not sure | 28 (4.5) |
| **Site of HP infection^†^** |  |
| Stomach (correct) | 544 (88.0) |
| Brain | 1 (0.2) |
| Heart | 1 (0.2) |
| Small intestine | 51 (8.3) |
| Kidney | 5 (0.8) |
| Liver | 3 (0.5) |
| Not sure | 46 (7.4) |
| **Route of transmission** |  |
| Hand and mouth | 379 (61.5) |
| Mother to child during pregnancy | 38 (6.2) |
| Sexual contact | 1 (0.2) |
| Injections | 0 (0) |
| All of the above | 25 (4.1) |
| Not sure | 173 (28.1) |
| No answer | 2(0.4) |
| **Household member transmission of HP** |  |
| Possible | 291 (47.2) |
| Not possible | 156 (25.3) |
| Not sure | 170 (27.6) |
| No answer | 1 (0.2) |
| **Diseases induced by HP** |  |
| Gastrointestinal (ulcer and cancer) | 583 (95.7) |
| Brain infection | 6 (1.0) |
| Pneumonia | 5 (0.8) |
| Kidney failure | 4 (0.7) |
| Liver cirrhosis | 3 (0.5) |
| No answer | 10 (2.0) |
| **Can screening detect HP?** |  |
| Yes | 529 (85.6) |
| No | 39 (6.3) |
| Not sure | 50 (8.1) |
| **Favorite type of examination for HP^†^** |  |
| Blood | 381 (61.7) |
| Stool | 80 (12.9) |
| Urinary | 237 (38.3) |
| Breath | 192 (31.1) |
| Endoscopy | 93 (15.0) |
| Refuse (do not want to be tested) | 11 (1.8) |
| **How is HP infection treated?** |  |
| Medicine | 557 (90.3) |
| Injections | 7 (1.1) |
| Surgery | 6 (1.0) |
| It can’t be treated | 5 (0.8) |
| Not sure | 42 (6.8) |
| No answer | 1 (0.2) |

**^†^**Multiple answers were provided.

**Supplementary Table 3. Acceptable tests for HP screening**

| **What type of test would you like to undergo for HP?** | **Number of answers (n=618)**  **n (%)** |
| --- | --- |
| Blood test | 381 (61.7) |
| Stool test | 80 (12.9) |
| Urinary test | 237 (38.3) |
| Breath test | 192 (31.1) |
| Endoscopy | 93 (15.0) |
| Do not want to undergo test | 11 (1.8) |

The numbers represent the number of answers and the proportion.

HP: *Helicobacter pylori*

**Supplementary table 4.** **Variables related to the desire for children’s HP screening**

|  | **Multivariable** |  |
| --- | --- | --- |
| **Variables** | **Adjusted OR (95% CI)** | ***P*-values** |
|  |  |  |
| **Knowledge of HP (reference = Insufficient, middle [0–4 point])** | | |
| Sufficient (5–7 points) | 2.98 (1.71 – 5.19) | <0.05 |
| **Relationship (reference = Father)** | |  |
| Mother | 1.52 (0.65 – 3.55) | 0.34 |
| Grandmother | 0.62 (0.05 – 7.05) | 0.70 |
| Grandfather | 0.94 (0.08 – 11.65) | 0.96 |
| **Age (reference = 30–39 y)** |  |  |
| 40–49 y | 2.23 (1.26 – 3.93) | <0.05 |
| 50 y– | 1.90 (0.81 – 4.48) | 0.14 |
| **Occupation (reference = Other jobs)** | |  |
| Medical professional | 1.62 (0.69 – 3.79) | 0.27 |
| **Family HP history (reference = Absent)** | |  |
| Present | 1.80 (0.84 – 3.86) | 0.13 |
| **Family cancer history (reference = Absent)** | |  |
| Present | 0.83 (0.46 – 1.50) | 0.54 |
| **HP screening history (reference = Absent)** | |  |
| Present | 0.66 (0.37 – 1.18) | 0.16 |
| **Cancer screening adherence (reference = Never)** | |  |
| Occasionally | 1.27 (0.71 – 2.26) | 0.43 |
| Annually | 1.64 (0.78 – 3.47) | 0.19 |

OR: odds ratio

CI: confidence interval

HP: *Helicobacter pylori*

**Supplementary Table 5a. Reasons for guardians supporting screening of children**

| **Reasons** | **n=534** |
| --- | --- |
| It’s a good opportunity | 284 (53.2) |
| Testing for HP is necessary | 235 (44) |
| The fee will be covered | 156 (29.2) |
| Family member has undergone tests for HP | 132 (24.7) |
| The screening is organized at school | 127 (23.8) |
| The other sibling underwent screening before | 106 (19.9) |
| We do not need to go hospital for the test | 103 (19.3) |
| Not specific | 17 (3.2) |
| Others | 14 (2.6) |
| Other children are willing to undergo screening | 3 (0.6) |

Multiple responses are obtained.

HP: *Helicobacter pylori*

**Supplementary Table 5b. Reasons for guardians not supporting the screening of their children**

| **Reasons** | **n=12** |
| --- | --- |
| Test for HP is not necessary | 8 (66.7) |
| My children don’t have HP | 1 (8.3) |
| I don’t understand what HP is. | 1 (8.3) |
| Few of our children’s friends are willing to undergo screening | 1 (8.3) |
| No family member has undergone testing for HP | 1 (8.3) |
| Others | 3 (24.9) |

Multiple responses are obtained.

HP: *Helicobacter pylori*
